# The Smk1 MAPK and Its Activator, Ssp2, Are Required for Late Prospore Membrane Development in Sporulating *Saccharomyces cerevisiae*

**DOI:** 10.3390/jof7010053

**Published:** 2021-01-14

**Authors:** Matthew Durant, Joseph M. Roesner, Xheni Mucelli, Christian J. Slubowski, Erin Klee, Brian C. Seitz, Zoey Wallis, Linda S. Huang

**Affiliations:** Department of Biology, University of Massachusetts Boston, 100 Morrisey Boulevard, Boston, MA 02125, USA; Matthew.Durant001@umb.edu (M.D.); joseph.roesner@merck.com (J.M.R.); xheni.mucelli001@umb.edu (X.M.); cslubowski@generationbio.com (C.J.S.); Erin.Klee001@umb.edu (E.K.); Brian.Seitz001@umb.edu (B.C.S.); wallisz@bc.edu (Z.W.)

**Keywords:** *Saccharomyces cerevisiae*, sporulation, prospore membrane, meiosis II, MAP kinase signaling, leading edge protein complex

## Abstract

During sporulation in the budding yeast *Saccharomyces cerevisiae*, proper development of the prospore membrane is necessary for the formation of viable spores. The prospore membrane will eventually become the plasma membrane of the newly formed haploid spore and also serves as the template for the deposition of the spore wall. The prospore membrane is generated de novo during meiosis II and the growing edge of the prospore membrane is associated with the Leading Edge Protein (LEP) complex. We find that the Smk1 MAP kinase, along with its activator Ssp2, transiently localizes with the LEP during late meiosis II. *SSP2* is required for the leading edge localization of Smk1; this localization is independent of the activation state of Smk1. Like other LEP components, the localization of Smk1 at the leading edge also depends on Ady3. Although prospore membrane development begins normally in *smk1* and *ssp2* mutants, late prospore membrane formation is disrupted, with the formation of ectopic membrane compartments. Thus, MAP kinase signaling plays an important role in the formation of the prospore membrane.

## 1. Introduction

Diploid *Saccharomyces cerevisiae* cells will form spores when starved of carbon and nitrogen (reviewed in [1]). The sporulation process involves meiosis followed by spore morphogenesis, resulting in the formation of four haploid spores from a diploid mother cell. The formation of spores requires a new membrane that begins growth during meiosis II; this membrane is called the prospore membrane. Cellularization of the haploid cells occurs when the prospore membrane grows to surround the haploid meiotic products.

The prospore membrane is critical for the development of the spore. The prospore membrane starts as a double lipid bilayer and serves as the template for spore wall deposition. Ultimately, the inner lipid bilayer becomes the plasma membrane of the new haploid cells. The outer bilayer is degraded during spore wall formation. The spore wall differs from the vegetative cell wall and is made up of four layers: the inner two layers are made of mannan and glucan, while the outer two layers are made up of chitosan and dityrosine.

Growth of the prospore membrane requires post-Golgi vesicles and begins at the spindle pole body [2,3,4]. The prospore membrane takes on characteristic shapes as it grows, starting as cups in early development, transitioning to elongated tube-like structures, and eventually rounding up and closing [5]. As the prospore membrane grows, a protein complex called the Leading Edge Protein (LEP) complex associates with the growing edge of the membrane. Members of the LEP include Ssp1, Don1, Ady3, and Irc10 [6,7,8,9,10]. The LEP localizes to the ring-shaped opening that forms as the prospore membrane grows around the nucleus and is thought to be necessary for the shape of the growing membrane and for keeping the lip of the membrane open as it grows [8,10,11]. The removal of this complex at the end of meiosis II is needed for the closure of the prospore membrane [5,6,12].

The Smk1 mitogen-activated protein kinase (MAPK) is required for spore wall formation [13,14]. *SMK1* negatively regulates the glucan synthase activity of Gsc2, an enzyme involved in the synthesis of the glucan layer of the spore wall [15]. *smk1∆* cells lack a chitosan layer [14,15], leading to the model that the chitosan layer may only form when glucan synthesis is properly shut off [15]. *SMK1* likely has other roles beyond the regulation of Gsc2, as *smk1* mutant cells do not form refractile spores [13,14,15], which differs from the *gsc2∆* mutants, which can form refractile compartments, even though spore wall formation is disrupted [15].

Conventional MAP kinases are activated by dual-specificity MAPK kinases (MAPKKs) that act as both a tyrosine and serine/threonine kinase. Smk1 activation does not require a typical MAPKK, even though Smk1 does have the conserved activation loop threonine (T) and tyrosine (Y). These conserved residues are important, as an in vitro kinase assay demonstrated that Smk1 requires phosphorylation at T207 and Y209 for its activity [15]. Instead of activation by a dual-specificity MAPKK, Smk1 activation requires both Ssp2 (which does not contain a protein kinase domain) and Cak1 (a serine/threonine protein kinase) [14,16,17]. Cak1 phosphorylates Smk1 at T207 while Ssp2 binding to Smk1 leads to autophosphorylation of Y209 [16,17].

*ssp2* mutants share some phenotypes with *smk1* mutants, suggesting these genes may play similar roles. *SSP2* is needed for spore wall formation [18,19] and *ssp2* mutants do not make the chitosan layer of the spore wall [19]. Smk1 and Ssp2 both localize to the prospore membrane during meiosis II [16,20], although defects in prospore membrane development have not been previously reported in *smk1* or *ssp2* mutants.

In this study, we show that Smk1 and Ssp2 have a transient localization at the leading edge of the growing prospore membrane late in meiosis II; this localization to the leading edge of the prospore membrane depends on *SSP2* and *ADY3* but not the activation state of Smk1. We demonstrate a role for *SMK1* and *SSP2* in late prospore membrane development, suggesting a role for MAP kinase signaling in this process.

## 2. Materials and Methods

### 2.1. Yeast Strains and Plasmids

All strains used in this study are in the SK1 background [21] and are described in Supplementary Table S1. All strains are derived from LH177 [15]. Standard genetic methods were used to create and propagate strains unless otherwise noted [22]. Epitope-tagged strains and knockout alleles were generated by PCR-mediated recombination techniques, as previously described [23,24,25].

The *pTEF1-SPO20^51–91^-mKate2::HIS3* allele used in this study was created using plasmid mod_pRS303-pTEF1-mKate2-SPO20^51–91^ [26], which was cut with *PstI* (New England Biolabs, Ipswich, MA, USA) and integrated at the *HIS3* locus. The *pTEF1-ENVY-SPO20^51–91^-tCYC1::URA3* allele used to examine prospore membranes was constructed by linearizing pRS426-E20 [27] with StuI and integrated at the *URA3* locus. The *pTEF1-ENVY-SPO20^51–91^::HIS3* allele was created by replacing *mKATE2* with *GFP^ENVY^* in mod_pRS303-pTEF1-mKate2-SPO20^51–91^ [26], to generate the resulting plasmid BSp56, which was verified by sequencing before cutting with Pst1 and integrated into the *HIS3* locus. The *pRS426-pTEF1-SPO20^51–91^-mTagBFP* plasmid used to label prospore membranes was previously described [28].

The plasmids containing the *smk1* activation loop mutations (CSp211, CSp212, CSp213) were constructed in pRS316 [29], a centromere-based low-copy plasmid. The activation loop mutations of *smk1* are expressed under the control of the *SMK1* promoter and have *GFP^ENVY^* at the C-terminus of the protein. Primers used to construct these mutations are listed in Table 1. The Smk1 promoter and part of *SMK1* was amplified using OLH1756 and OLH1757, while the remainder of the *SMK1* gene was amplified using OLH1773 and OLH1774 while also providing overlap with the linker sequence for the insertion of *GFP^ENVY^*, which was amplified using OLH1758 and OLH1759. OLH1756 created an SacI site at the 5′ end, while OLH1759 created a XhoI site at the 3′ end of the construct, allowing for the assembly of the *SMK1* locus in pRS316. DNA fragments containing the T207A, Y209F, and T207 Y209F changes were obtained from plasmids carrying these mutations [15] using the naturally occurring BglII and HindIII site within the *SMK1* locus. Plasmids were sequenced to verify appropriate construction.

Cells were grown in standard yeast media and sporulated in a synchronous manner as previously described [15]. Briefly, yeast cells were grown in YPD (yeast extract—peptone—dextrose, Thermofisher, Waltham, MA, USA) overnight at 30 °C and then transferred to YPAcetate (yeast extract—peptone–potassium acetate, Thermofisher, Waltham, MA, USA) and grown to ~1.5 O.D_600_/mL overnight. These cells were collected, washed in ddH_2_O and resuspended in 1% potassium acetate at 2.0 O.D_600_/mL.

### 2.2. Immunoblotting

Cells were collected after 6 h in sporulation media and prepared using the trichloroacetic acid (TCA) precipitation method [30], which involves the addition of lysis buffer (1.85N NaOH, 10% *v*/*v* β-mercaptoethanol, Sigma-Aldrich, St. Louis, MO, USA), followed by precipitation of proteins with 50% (*v*/*v*) TCA. Precipitated protein lysates are then pelleted, washed with ice-cold acetone, and resuspended in 2× sample buffer (20% glycerol (*w*:*v*), 2% SDS (*w*:*v*), 2% β-mercaptoethanol (*v*:*v*), 0.05% bromophenol blue (*w*:*v*), 62.5 mM Tris-HCl pH 6.8, Thermofisher, Waltham, MA, USA) neutralized with 5 µL 1 M Tris base; samples were boiled before loading. Protein lysates were separated on an 8% SDS-PAGE gel.

Separated protein extracts were then transferred onto Immobilon low-fluorescence PVDF membrane, blocked with TBS blocking buffer (LI-COR, Lincoln, NE, USA), and incubated with appropriate primary antibodies. HA was detected using anti-HA.11 16B12 antibody (901501, Biolegend, San Diego, CA, USA) at a 1:2000 dilution. Pgk1 was detected using a rabbit polyclonal anti-Pgk1 antibody (PA5-28612, ThermoFisher, Waltham, MA, USA) at a 1:5000 dilution. Membranes were imaged using a LI-COR Odyssey Infrared Imaging System, using IRDye 800CW anti-mouse secondary antibodies and IRDye680RD anti-rabbit secondary antibodies at a 1:10,000 dilution (LI-COR, Lincoln, NE, USA).

### 2.3. Microscopy

Widefield microscopy was performed using a 100× (NA 1.45) objective on an Axioskop Mot2 (Zeiss, White Plains, NY, USA). Images were taken using an Orca-ER cooled CCD camera (Hamamatsu) using iVision (BioVision Technologies, Exton, PA, USA) software for image acquisition. Cells were optically sectioned in the Z-dimension in each channel. Z-sections were then merged using max intensity projection, adjusted for equivalent brightness and contrast, pseudocolored, merged, and cropped using FIJI [31].

## 3. Results

### 3.1. Smk1 Localizes to the Leading Edge of Elongated Prospore Membranes

**Figure 1:**
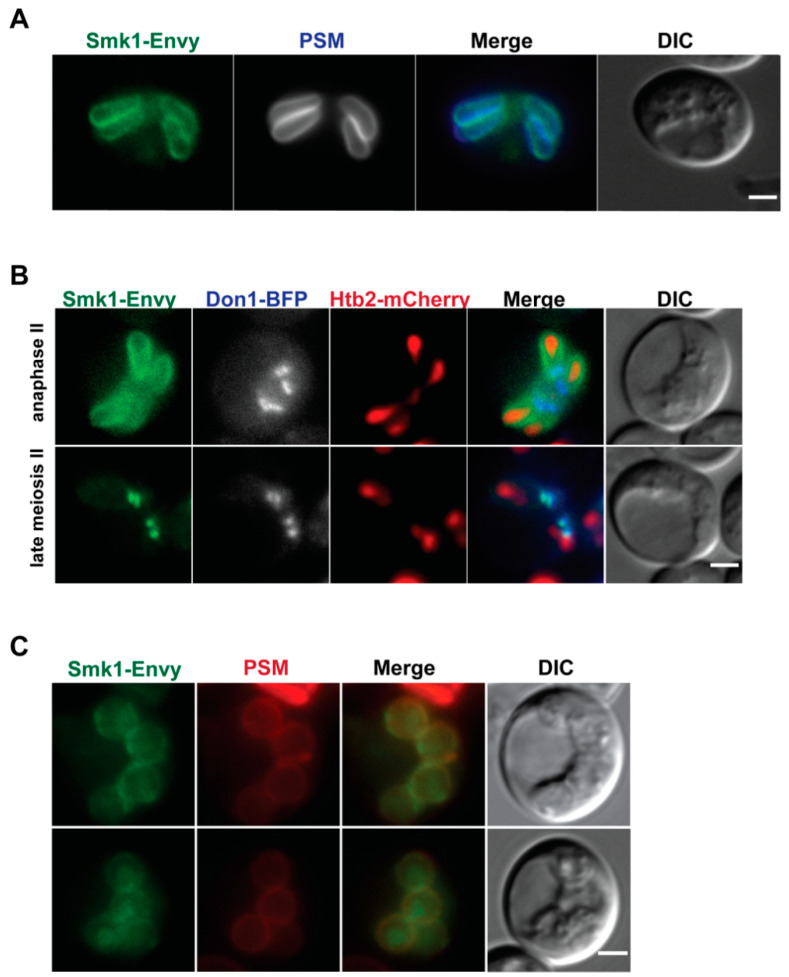
Smk1 exhibits two distinct localizations during prospore membrane development. (**A**) Smk1-GFP^Envy^ co-localizes with the prospore membrane (PSM) marker pRS426-Spo20^51–91^-mTagBFP along the PSM. (**B**) Smk1-GFP^Envy^ localizes to PSM during the elongation stage of membrane development, and is concentrated at the leading edge of the prospore membrane after anaphase II. Htb2-mCherry is used as a histone marker. The leading edge of the growing prospore membrane is marked using Don1-BFP. (**C**) Smk1-GFP^Envy^ becomes more broadly localized after prospore membranes round up and close. Prospore membranes are in red and are labeled using an integrated Spo20^51–91^-mKate2 marker. For all strains, the genomic copy of *SMK1* is epitope tagged with *GFP^ENVY^* at its C-terminus; strains are homozygous for *SMK1-GFP^ENVY^*. Yeast strains used for each panel: (**A**) LH1116, (**B**), LH1117, and (**C**) LH1133. Scale bar = 2 µM.

Previous studies that report Smk1 localization to the prospore membrane utilized an overexpressed version of Smk1-GFP, due to difficulties in imaging endogenous levels of Smk1 [16]. Because GFP^ENVY^ is brighter and more photostable [25], we made a genomically integrated *SMK1-GFP^ENVY^*allele and examined Smk1 localization during sporulation. As previously reported, we see localization at the prospore membrane during meiosis II (Figure 1A and 1B top) and co-localization of the Smk1-GFP^ENVY^ signal with a marker for the prospore membrane that fuses a fluorescent protein to a lipid-binding domain from the Spo20 protein (residues 51–91) [32].

During late meiosis II, as assayed by examining the prospore membrane morphology and by following meiosis using the nuclear marker Htb2-mCherry, we see transient concentrated localization of Smk1 (Figure 1B bottom). These regions of concentrated Smk1 co-localize with Don1, a member of the LEP (Figure 1B bottom), showing that Smk1 localizes to the leading edge of the prospore membrane during late meiosis II. Once PSMs round up and close, Smk1 becomes more diffuse and can be seen more broadly in the cell, where it can be found in the cytosol, along the prospore membrane, as well as in the nucleus (Figure 1C). Thus, Smk1 localization is dynamic, starting all along the prospore membrane, becoming concentrated at the leading edge after anaphase II, and becoming more broadly localized after prospore membranes round and close.

### 3.2. Smk1 Localization at the Leading Edge of the Prospore Membrane Requires SSP2

**Figure 2:**
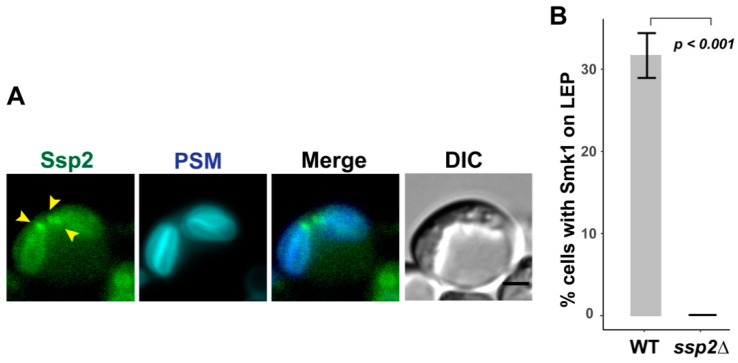
*SSP2* is required for Smk1 localization at the leading edge of the prospore membrane. (**A**) Ssp2 localizes to the leading edge of the prospore membrane. Ssp2-GFP^Envy^ is found at the leading edge of the elongated prospore membrane (LH1118). Prospore membranes (PSMs) are labeled with pRS426-Spo20^51–91^-mTagBFP. Yellow arrowheads indicate leading edge localization at the open ends of the tubular elongated prospore membranes. Scale bar = 2 µM. (**B**) Percentage of cells in late meiosis II that have Smk1 localized at the leading edge of the prospore membrane. Wild type (WT; LH1117), *ssp2∆* (LH1134). Three biological replicates, 100 cells per replicate, were counted for each genotype. Error bars show standard error of the mean. *p*-value was calculated using Fisher’s exact test (two-tailed).

Previous studies that demonstrated Ssp2 localization on the prospore membrane also utilized an overexpressed version of Ssp2 [16]. Because Ssp2 complexes with Smk1, we examined whether Ssp2 was also localized at the leading edge of the prospore membrane. We created a genomically integrated *SSP2-GFP^ENVY^* allele and see that in addition to the previously reported localization along the prospore membrane, Ssp2 can also localize to the leading edge of the prospore membrane late in meiosis II, when prospore membranes are elongated (Figure 2A). Thus, the localization of Smk1 and Ssp2 are similar during meiosis II.

We tested whether the localization of Smk1 at the leading edge required *SSP2*. We compared the localization of Smk1-GFP^ENVY^ in wild type cells and *ssp2∆* cells. We only counted cells in late meiosis II, as assayed by using Htb2-mCherry and Don1-BFP to identify cells with four distinct nuclei that still had Don1-BFP localized as a tight ring or dot (like that seen in Figure 1B bottom). We found that ~30% of these cells had Smk1 concentrated along the leading edge of the prospore membranes (Figure 2B). Cells that did not have Smk1 at the leading edge had Smk1 localized along the prospore membrane. We hypothesize that the reason we do not see 100% of wild type cells having Smk1 at the leading edge is due to the transient nature of the leading edge localization coupled with the imperfect synchrony seen in sporulating SK1 cells; when we have used genetic methods to synchronize sporulation, the best we have seen is 50% of cells having Smk1 along the leading edge in the otherwise wild type culture (unpublished observations).

In contrast, we do not see Smk1 localizing at the leading edge of the growing prospore membrane in *ssp2∆* cells in late meiosis II (as assayed using Htb2-mCherry and Don1-BFP) (Figure 2B). Instead, 100% of the *ssp2∆* cells in late meiosis II had Smk1 localized along the prospore membrane. Thus, although the localization of Smk1 on the prospore membrane is independent of *SSP2* (as previously reported [16]), its localization at the leading edge requires *SSP2*.

### 3.3. Smk1 Localization Is Independent of Activation State

Because Ssp2 activates Smk1, we asked whether localization of Smk1 at the leading edge of the prospore membrane depended on its activation state. We created *GFP^ENVY^*-tagged versions of *smk1-T207A*, *smk1-Y209F*, and *smk1-T207A Y209F*, mutating the conserved threonine (T) and tyrosine (Y) in the conserved activation loop to the non-phosphorylateable amino acids alanine (A) and phenylalanine (F).

**Figure 3:**
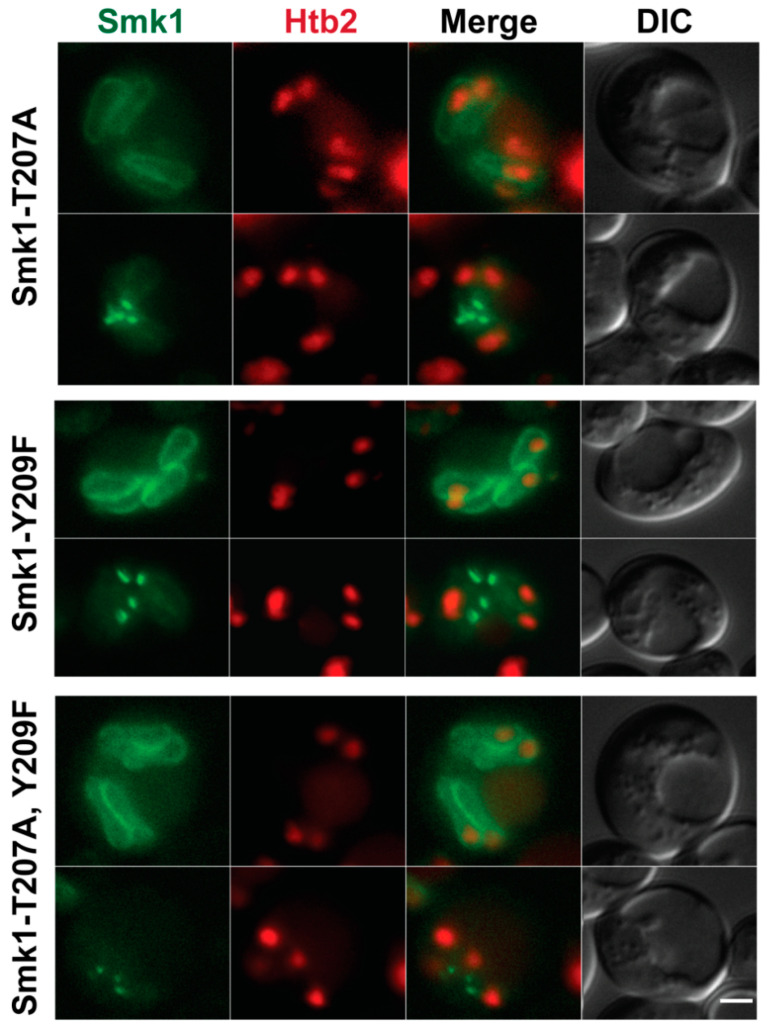
Smk1 localization is independent of its activation state. Localization of Smk1-T207A (LH1119), Smk1-Y209F (LH1120), and Smk1-T207A, Y209F (LH1121) to both elongated prospore membranes and the leading edge of the prospore membrane. *smk1* activation site mutations are epitope tagged with *GFP^ENVY^*. Htb2-mCherry is used as a histone marker. Scale bar = 2 µM.

We examined the localization of the Smk1-T207A, Smk1-Y209F, and Smk1-T207A Y209F proteins during meiosis II and found that all three of these inactivation mutants of Smk1 can still localize along the prospore membrane during most of meiosis II and also concentrates at the leading edge towards the end of meiosis II (Figure 3). These results suggest that the ability of Smk1 to localize at the prospore membrane and the leading edge is independent of the phosphorylation state of its activation loop and also of its activity, as these mutations inactivate the Smk1 kinase [15].

### 3.4. Localization of Smk1 Requires the LEP Member ADY3, But Not IRC10 or DON1

**Figure 4:**
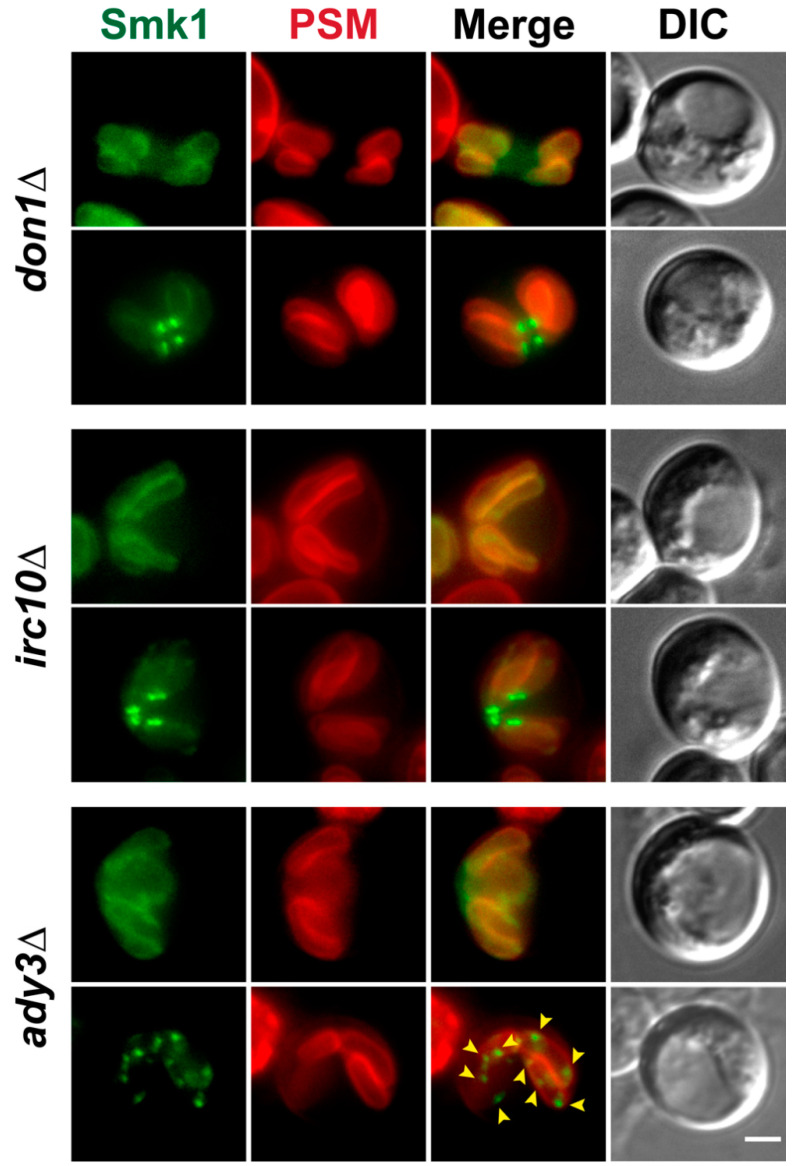
Smk1 localization requires the Leading Edge Protein complex member *ADY3*, but not *IRC10* or *DON1*. Smk1-GFP^ENVY^ is localized to the prospore membrane and the leading edge in *don1∆* (LH1123) and *irc10∆* (LH1124) mutants. Smk1-GFP^ENVY^ is localized to the prospore membrane (PSM) in *ady3∆* (LH1125) mutants early in meiosis II, but becomes localized as puncta along the leading edge later in development. Prospore membranes are in red and are labeled using an integrated Spo20^51–91^-mKate2 marker. Yellow arrowheads indicate some of the ectopic Smk1 puncta seen along the prospore membrane in *ady3*∆ mutant cells. Scale bar = 2 µM.

Because Smk1 co-localizes with the LEP, we tested whether Smk1 localization at the leading edge depended on *IRC10* or *DON1*, two members of the LEP. Like in wild type cells, in *irc10∆* or *don1∆* cells, Smk1 can be seen on the prospore membrane during most of meiosis II, including at the elongated tubular stage of prospore membrane development (Figure 4, top panel of each genotype). Late in meiosis, after anaphase II, Smk1 can be seen concentrated at the leading edge in *irc10∆* and *don1∆* cells (Figure 4, bottom panel of each genotype). These results show that the localization of Smk1 along the prospore membrane and at the leading edge does not require *IRC10* or *DON1*.

*ADY3* is a member of the LEP and is important for the localization of other LEP components [8,9,10]. When we examine Smk1 localization in cells lacking *ADY3*, we see that during most of meiosis II, Smk1 localizes to the prospore membrane, as in wild type cells (Figure 4). However, during late meiosis II (when prospore membranes are elongated), at the time when Smk1 would co-localize with the leading edge protein Don1 (Figure 1B), we instead see Smk1 localizing to puncta along the prospore membrane in *ady3*∆ mutants (Figure 4). Thus, although *ADY3* is dispensable for the localization of Smk1 during most of meiosis II, *ADY3* is required for appropriate Smk1 localization during late prospore membrane development.

### 3.5. Smk1 Phosphorylation Does Not Require the LEP Complex

Smk1 is phosphorylated around 6 h during sporulation; this phosphorylation can be seen on immunoblots as a more slowly migrating band. This more slowly migrating band is missing when Smk1 first appears during sporulation (~5 h) and appears around the time when cell are in anaphase II (~6 h) [15].

**Figure 5:**
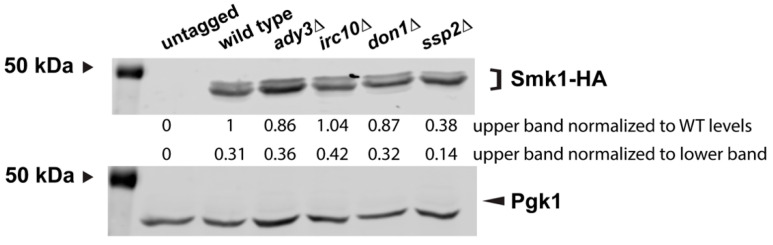
Smk1 post-translational modification does not require the leading edge protein complex members *ADY3*, *IRC10*, and *DON1*. Immunoblot of protein extracts examining Smk1-HA in an untagged strain lacking Smk1-HA (LH177), Smk1-HA (LH178), *ady3∆* (LH1126), *irc10∆* (LH1127), *don1∆* (LH1128), and *ssp2∆* (LH1129). Pgk1 is used as a loading control. The 50 kDa band from the Precision Plus Protein Markers (BioRad) is indicated on the left of each blot. The upper quantification corresponds to densitometry, in which the slower migrating (upper) Smk1-HA band was first normalized to Pgk1 levels, followed by the normalization of the slower migrating Smk1-HA band in each mutant to wild-type levels of expression. The lower quantification corresponds to densitometry, in which the slower migrating Smk1-HA band was normalized to the faster migrating Smk1-HA band for each genotype.

To see whether the LEP is required for the post-translational modification of Smk1, we examined cells 6 h into sporulation when the characteristic doublet can be easily seen, using the *SMK1-HA* allele. We do not see significant differences in Smk1 post-translational modification in *irc10∆*, *don1∆*, *ssp2∆*, or *ady3∆* mutants (Figure 5). As expected, we do see a slight decrease in the more slowly migrating Smk1 band in *ssp2∆* mutants, consistent with *SSP2* being required for the full phosphorylation and activation of Smk1 [16].

### 3.6. SMK1 and SSP2 Are Required for Late Prospore Membrane Development

**Figure 6:**
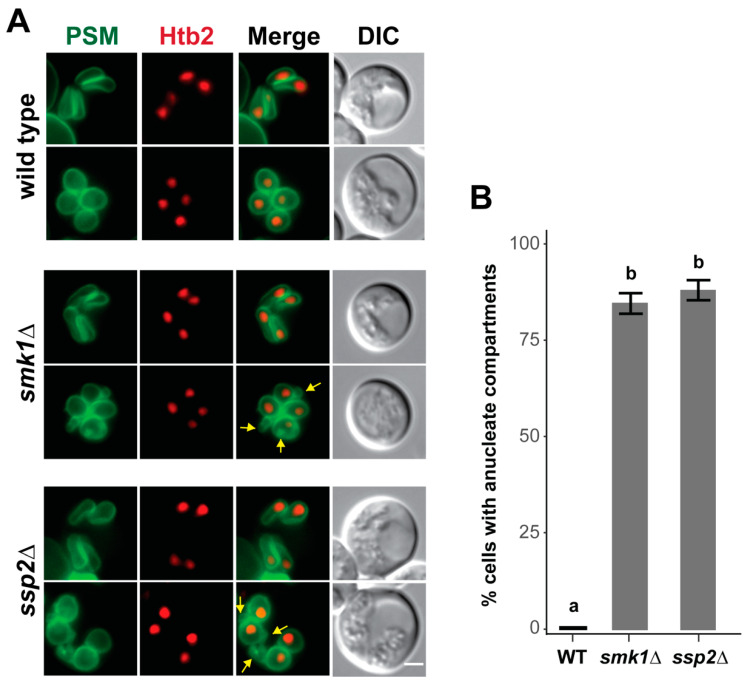
*SMK1* and *SSP2* are required for late prospore membrane development. (**A**) Cells with elongated tubular prospore membranes and post-anaphase II rounded prospore membranes are shown in wild type (LH1130), *smk1∆* (LH1131), and *ssp2∆* (LH1132) cells. Prospore membranes (PSM) are in green and are labeled using an integrated Spo20^51–91^-GFP^ENVY^ marker. Htb2-mCherry was used as a histone marker in LH1131 and LH1132, while Htb2-mRuby2 was used as the histone marker in LH1130. Yellow arrows point to ectopic membrane compartments found in mutant cells with rounded prospore membranes. Scale bar = 2 µM. (**B**) Quantitation of anucleate compartments. Post-anaphase II cells that had four distinct nuclei and rounded prospore membranes were assayed for the presence of anucleate compartments in wild type (WT; LH1130), *smk1∆* (LH1131), and *ssp2∆* (LH1132). At least three biological replicates, 100 cells per replicate, were counted for each genotype. Error bars show the standard error of the mean. The wild type strain (a) is significantly different from the *smk1∆* and *ssp2∆* strains (b); one way ANOVA (F(2,8) = 549, *p* < 0.001) followed by Tukey’s HSD post hoc test (alpha = 0.01).

Given the localization of Smk1 and Ssp2 to the leading edge of the prospore membrane in late meiosis II, we asked whether prospore membrane development was disrupted in *smk1∆* or *ssp2∆* cells. As previously reported, we do not see obvious prospore membrane defects in the early stages of prospore membrane during meiosis up to anaphase II, when prospore membrane form elongated tube-like structures (Figure 6).

However, at the end of meiosis II, when prospore membranes round up and close, we see additional membrane compartments in the *smk1∆* and *ssp2∆* mutant cells (Figure 6A). These extra compartments are anucleate and are only seen late in prospore membrane development after anaphase II, when the four meiotic nuclei are distinctly separated. These anucleate compartments are seen in >85% of *smk1∆* and *ssp2∆* cells with rounded prospore membranes, and are not found in wild type cells (Figure 6B). Thus, although *SMK1* and *SSP2* are dispensable during most of meiosis II, late prospore membrane development does not occur properly in the absence of these genes.

## 4. Discussion

Here, we find a role for the Smk1 MAPK and its activator, Ssp2, in late prospore membrane development. Using an integrated GFP^ENVY^-tagged Smk1, we see the transient localization of Smk1 and Ssp2 at the leading edge of the prospore membrane after anaphase II, before cellularization when the prospore membrane rounds up and closes. This leading edge localization is transient and can only be seen in cells at the very end of meiosis II.

Previous studies that had examined Smk1 and Ssp2 only reported the earlier occurring prospore membrane localization [16,20]. These studies used an overexpression plasmid due to difficulties in detecting endogenous levels of Smk1. GFP^ENVY^ is brighter and more photostable than the more widely used eGFP [25], which allowed for the visualization of Smk1 expressed from the genomic locus under its own promoter at two copies per cell. It is possible that the leading edge localization was previously missed, either because the overproduction of Smk1 led to its localization at both the prospore membrane and the leading edge in late meiosis II, which would obscure the leading edge localization, or possibly because the leading edge localization is transient and can only be caught at a brief window at the end of meiosis II (meiosis II occurs relatively rapidly within about 40–50 min and anaphase II only lasts 10–20 min [33].)

Our current results do not allow us to distinguish between models of where the Smk1 and Ssp2 at the leading edge originates. Neither Smk1 nor Ssp2 are predicted to be transmembrane proteins, and thus, are likely peripheral membrane proteins; a membrane binding domain has been identified at the N-terminus of Ssp2 [16]. It is possible that the Smk1 and Ssp2 that is concentrated at the leading edge comes from newly recruited pools from the cytoplasm. Alternatively, it is possible that Smk1 and/or Ssp2 is relocalized from the pool of Smk1 and Ssp2 already on the prospore membrane.

We find that the LEP component *ADY3* is required for the proper recruitment of Smk1 to the leading edge of the prospore membrane, even though it is not required for the initial localization of Smk1 to the prospore membrane. Interestingly, the punctate localization of Smk1 on the prospore membrane in *ady3*∆ mutants is reminiscent of the mislocalization of the LEP component Ssp1 seen in *ady3∆* and *ady3∆ irc10∆* mutants [8,10]. The consequence of these mislocalized proteins is unclear, as *ady3∆* mutants can form mature spores (unlike *ssp1∆* or *smk1∆*), although *ady3∆* mutants have a tendency to form asci with only two spores [8,9].

We also see that *SSP2*, which encodes the activator of Smk1, is required for the proper recruitment of Smk1 to the leading edge of the prospore membrane. The earlier Smk1 localization to the prospore membrane occurs independently of *SSP2*. Ssp2 complexes with Smk1 [16] and shares the same localization, suggesting that Smk1 activation could occur in a localized fashion. However, a mutant that lacks the Ssp2 membrane binding domain at its N-terminus (*ssp2-∆N137)* sporulates normally [16], suggesting that Smk1 activation cannot only occur at the prospore membrane. We see that Smk1 localization to the prospore membrane and the leading edge is independent of its activation state, as mutations that prevent activation do not perturb the localization of Smk1. Thus, *SSP2* may play a role in the movement and/or recruitment of inactive Smk1 to the leading edge, in addition to its role in mediating Smk1 autophosphorylation on Y209. It is possible that Smk1 and Ssp2 are recruited to the prospore membrane independently. Whether the mechanism that relocalizes Ssp2 to the leading edge also brings along Smk1 as a member of the complex, or whether Ssp2 is directly responsible for the movement/recruitment of Smk1 once Ssp2 arrives at the leading edge and does not complex with Smk1 until they are both at the leading edge, still needs to be determined.

Because the removal of the LEP at the end of meiosis II is important for prospore membrane closure, we initially hypothesized a role in closure for *SMK1* and *SSP2*. However, the *smk1∆* and *ssp2∆* prospore membrane phenotype is distinct from the defect seen in mutants such as *ama1∆*, *cdc15∆*, *spo77∆*, and *sps1∆*, which play a role in prospore membrane closure [5,12,27]. These mutants make hyperelongated prospore membranes that round up and close much later than wild type cells.

Instead, in *smk1∆* and *ssp2∆* mutants, we observed the formation of ectopic anucleate membrane compartments in post-anaphase II cells. These extra prospore membrane compartments may correspond to the ectopic membrane compartments previously seen by electron microscopy in the *ssp2∆* mutants [20]. Whether the prospore membrane phenotype is related to the localization of Smk1 and Ssp2 at the leading edge of the prospore membrane remains to be determined. How these extra membrane compartments form is also unclear: are cells closing the prospore membrane in the wrong place, creating the extra compartments, or are these extra compartments the result of inappropriate membrane synthesis after prospore membrane closure? A further examination of how *SMK1* and *SSP2* affect prospore membrane development can be a starting point for learning more about how MAP kinase signaling can affect membrane development.

## Figures and Tables

**Table 1:** Primers used to construct *smk1* activation loop mutations.

Primer Name	Sequence (5′ to 3′)
OLH1756	AATCGAGCTCAGTAATAAATACTGTGTTGTTTG
OLH1757	GAAAATGTTGGACCATG
OLH1773	GTACTCCTGATAAAGATATTCTG
OLH1774	CAGCACCGTCACCTAAAGACGAGGAGGACAAATC
OLH1758	TATAAAAAAATCCCATGGTC
OLH1759	ATTGCTCGAGTTATTTGTACAATTCGTCCATTC

## Data Availability

Not applicable.

## Supplementary Materials

The following are available online at https://www.mdpi.com/2309-608X/7/1/53/s1, Table S1: Yeast Strains.
