# Supplementary material for: The Smk1 MAPK and Its Activator, Ssp2, Are Required for Late Prospore Membrane Development in Sporulating Saccharomyces cerevisiae
Source: J Fungi (Basel). 2021 Jan 14;7(1):53. doi: 10.3390/jof7010053 (PMC7828665; doi:10.3390/jof7010053)
Supplement: Supplementary file 1 [file jof-07-00053-s001.zip › Durant Roesner Table S1.pdf]

TABLE S1: Yeast Strains (page 1 of 2)

Strain	Genotype	Source
LH177	*MATa/MATα ho::LYS2/ho::LYS2 lys2/lys2 ura3/ura3 leu2/leu2 his3/his3 trp1ΔFA/trp1ΔFA*	[15]
LH178	*MATa/MATα ho::LYS2/ho::LYS2 lys2/lys2 ura3/ura3 leu2/leu2 his3/his3 trp1ΔFA/trp1ΔFA SMK1::3xHA-HIS3MX6/SMK1-3xHA-HIS3MX6*	[15]
LH1113	*MATa/MATα ho::LYS2/ho::LYS2 lys2/lys2 ura3/ura3 leu2/leu2 his3/his3 trp1ΔFA/trp1ΔFA SSP2:ENVY:HIS3*$^{S.p}$*/SSP2:ENVY:HIS3*$^{S.p}$	This study
LH1114	*MATa/MATα ho::LYS2/ho::LYS2 lys2/lys2 ura3/ura3 leu2/leu2 his3/his3 trp1ΔFA/trp1ΔFA SMK1::ENVY-HIS3MX6/SMK1-ENVY-HIS3MX6*	This study
LH1115	*MATa/MATα ho::LYS2/ho::LYS2 lys2/lys2 ura3/ura3 leu2/leu2 his3/his3 trp1ΔFA/trp1ΔFA SMK1:ENVY:HIS5/SMK1:ENVY:HIS5 ssp2::LEU2/ssp2::LEU2*	This study
LH1116	LH1114 plus *pRS426-pTEF2-SPO20*$^{51\text{-}91}$*-mTagBFP*	This study
LH1117	*MATa/MATα ho::LYS2/ho::LYS2 lys2/lys2 ura3/ura3 leu2/leu2 his3/his3 trp1ΔFA/trp1ΔFA SMK1:ENVY:HIS5/SMK1:ENVY:HIS5 DON1:BFP:KAN/DON1:BFP:KAN HTB2:mCherry:URA3/HTB2:mCherry:URA3*	This study
LH1118	LH1113 plus *pRS426-pTEF2-SPO20*$^{51\text{-}91}$*-mTagBFP*	This study
LH1119	LH1122 plus *pRS316-pSMK1-smk1(T207A)-ENVY* (CSp211)	This study
LH1120	LH1122 plus *pRS316-pSMK1-smk1(Y209F)-ENVY* (CSp212)	This study
LH1121	LH1122 plus *pRS316-pSMK1-smk1(T207AY209F)-ENVY* CSp213)	This study
LH1122	*MATa/MATα ho::LYS2/ho::LYS2 lys2/lys2 ura3/ura3 leu2/leu2 his3/his3 trp1ΔFA/trp1ΔFA smk1::LEU2/smk1::LEU2 HTB2:mCherry:TRP1/HTB2-mCherry:TRP1*	This study
LH1123	*MATa/MATα ho::LYS2/ho::LYS2 lys2/lys2 ura3/ura3 leu2/leu2 his3ΔSK::pTEF1-mKate2-SPO20*$^{51\text{-}91}$*::HIS3/his3ΔSK::pTEF1-mKate2-SPO20*$^{51\text{-}91}$*::HIS3 trp1ΔFA/trp1ΔFA don1::TRP1*$^{C.g}$*/ don1::TRP1*$^{C.g}$ *SMK1::ENVY:HIS3*$^{S.p}$*/SMK1::ENVY:HIS3*$^{S.p}$	This study
LH1124	*MATa/MATα ho::LYS2/ho::LYS2 lys2/lys2 ura3/ura3 leu2/leu2 his3ΔSK::pTEF1-mKate2-SPO20*$^{51\text{-}91}$*::HIS3/his3ΔSK::pTEF1-mKate2-SPO20*$^{51\text{-}91}$*::HIS3 trp1ΔFA/trp1ΔFA irc10::LEU2/ irc10::LEU2 SMK1::ENVY:HIS3*$^{S.p}$*/SMK1::ENVY:HIS3*$^{S.p}$	This study
LH1125	*MATa/MATα ho::LYS2/ho::LYS2 lys2/lys2 ura3/ura3 leu2/leu2 his3ΔSK::PTEF1-mKate2-SPO20*$^{51\text{-}91}$*::HIS3/his3ΔSK::pTEF1-mKate2-SPO20*$^{51\text{-}91}$*::HIS3 trp1ΔFA/trp1ΔFA ady3::TRP1*$^{C.g}$*/ ady3::TRP1*$^{C.g}$ *SMK1::ENVY:HIS3*$^{S.p}$*/SMK1::ENVY:HIS3*$^{S.p}$	This study
LH1126	*MATa/MATα ho::LYS2/ho::LYS2 lys2/lys2 ura3/ura3 leu2/leu2 his3/his3 trp1ΔFA/trp1ΔFA SMK1::3xHA-HIS3MX6/SMK1-3xHA-HIS3MX6 ady3::TRP1*$^{C.g}$*/ ady3::TRP1*$^{C.g}$ *HTB2:mCherry:TRP1*$^{C.g.}$*/HTB2:mCherry:TRP1*$^{C.g.}$	This study
LH1127	*MATa/MATα ho::LYS2/ho::LYS2 lys2/lys2 ura3/ura3 leu2/leu2 his3/his3 trp1ΔFA/trp1ΔFA SMK1::3xHA-HIS3MX6/SMK1-3xHA-HIS3MX6 irc10::LEU2/irc10::LEU2 HTB2:mCherry:TRP1*$^{C.g.}$*/HTB2:mCherry:TRP1*$^{C.g.}$	This study
LH1128	*MATa/MATα ho::LYS2/ho::LYS2 lys2/lys2 ura3/ura3 leu2/leu2 his3/his3 trp1ΔFA/trp1ΔFA SMK1::3xHA-HIS3MX6/SMK1-3xHA-HIS3MX6 don1::TRP1*$^{C.g}$*/ don1::TRP1*$^{C.g}$ *HTB2:mCherry:TRP1*$^{C.g.}$*/HTB2:mCherry:TRP1*$^{C.g.}$	This study
LH1129	*MATa/MATα ho::LYS2/ho::LYS2 lys2/lys2 ura3/ura3 leu2/leu2 his3/his3 trp1ΔFA/trp1ΔFA SMK1::3xHA-HIS3MX6/SMK1-3xHA-HIS3MX6 ssp2::LEU2/ssp2::LEU2 HTB2:mCherry:TRP1*$^{C.g.}$*/HTB2:mCherry:TRP1*$^{C.g.}$	This study

TABLE S1, page 2 of 2

Strain	Genotype	Source
LH1130	*MAT**a**/MATα ho::LYS2/ho::LYS2 lys2/lys2 ura3::pTEF1-ENVY-SPO20$^{51-91}$-tcyc::ura3/ ura3::pTEF1-ENVY-SPO20$^{51-91}$-tcyc::ura3 leu2/leu2 his3/his3 trp1ΔFA/trp1ΔFA HTB2-mRUBY2:URA3/HTB2-mRUBY2:URA3*	This study
LH1131	*MAT**a**/MATα ho::LYS2/ho::LYS2 lys2/lys2 ura3::pTEF1-ENVY-SPO20$^{51-91}$-tcyc::ura3/ ura3::pTEF1-ENVY-SPO20$^{51-91}$-tcyc::ura3 leu2/leu2 his3/his3 trp1ΔFA/trp1ΔFA smk1::LEU2/smk1::LEU2 HTB2:mCherry:TRP1$^{C.g./}$HTB2:mCherry:TRP1$^{C.g.}$*	This study
LH1132	*MATa/MATα ho::LYS2/ho::LYS2 lys2/lys2 his3ΔSK::pTEF1-ENVY-SPO20$^{51-91}$::HIS3/his3ΔSK::pTEF1-ENVY-SPO20$^{51-91}$::HIS3 leu2/leu2 his3/his3 trp1ΔFA/trp1ΔFA ssp2::LEU2/ssp2::LEU2 HTB2:mCherry:TRP1$^{C.g./}$HTB2:mCherry:TRP1$^{C.g.}$*	This study
LH1133	*MAT**a**/MATα ho::LYS2/ho::LYS2 lys2/lys2 ura3/ura3 leu2/leu2 his3/his3 trp1ΔFA/trp1ΔFA SMK1::ENVY-HIS3MX6/SMK1-ENVY-HIS3MX6 his3ΔSK::pTEF1-mKate2-SPO20$^{51-91}$::HIS3/his3ΔSK::pTEF1-mKate2-SPO20$^{51-91}$::HIS3*	This Study
LH1134	*MAT**a**/MATα ho::LYS2/ho::LYS2 lys2/lys2 ura3/ura3 leu2/leu2 his3/his3 trp1ΔFA/trp1ΔFA SMK1:ENVY:HIS5/SMK1:ENVY:HIS5 DON1:BFP:KAN/DON1:BFP:KAN HTB2:mCherry:URA3/HTB2:mCherry:URA3 ssp2::LEU2/ssp2::LEU2*	This study
